# Historical Highlight: The Luria-Delbrück Fluctuation Test – A Study of the Nature of Bacterial Mutations Conferring Resistance to Infection by Bacteriophage

**DOI:** 10.20411/pai.v10i1.763

**Published:** 2024-10-17

**Authors:** Neil S. Greenspan, Emily N. Kukan

**Affiliations:** 1 Case Western Reserve University, Cleveland, OH

**Keywords:** Bacteriophage, resistance to infection, mutation, mutation rate, statistical variance

In 1943, Salvador Luria, then at Indiana University, and Max Delbrück, then at Vanderbilt, published an analysis of mutations in *Escherichia coli* conferring resistance to infection by bacterial viruses, also referred to as bacteriophages [1]. Of note, Luria and Delbrück advanced our understanding of mutation prior to the publication by Oswald Avery, Colin MacLeod, and Maclyn McCarty in 1944 demonstrating that the so-called transforming principle, which was able to dramatically alter the surface and functional phenotypes of pneumococci, was composed of DNA [2]. The Avery et al paper is the focus of the initial Historical Highlight [3].

As we discuss below, in light of relatively recent discoveries relating to mechanisms that can be deployed by bacteria to resist bacteriophages, the identity of the particular *E. coli* strain Luria and Delbrück used is of interest. Had they employed a different bacterial strain, their results might have been more difficult to interpret.

The problem that Luria and Delbrück chose to investigate focused on two hypotheses about the mechanism by which mutations in bacteria, such as *E. coli*, confer resistance to bacterial viruses: 1) the viruses act directly on the bacteria they encounter to elicit or activate the resistance mechanism, or 2) the resistance depends on pre-existing genetic change (ie, mutation), and the viruses effectively select for those bacteria bearing a relevant mutation by killing the sensitive bacteria.

Specifically, Luria and Delbrück used a mathematical analysis of the differing expectations for variation in the numbers of resistant mutants in relatively small parallel cultures to determine which of the two alternative hypotheses was superior. This focus on the variation in numbers of resistant cells observed at a single time point after the initiation of the cultures, which was revealed following exposure to the virus, is why the method is sometimes referred to as the “fluctuation test.”

The authors pursued this mathematical approach because they believed it had the power to clearly support only one of the alternative hypotheses. In other words, they believed it was an incisive experimental test. If resistance to phage attack was based on spontaneously occurring and heritable mutations (phenotypic changes that are reliably transmitted to progeny cells) before any exposure to virus, then small parallel cultures of the bacteria should vary widely in the number of resistant cells found when the cultures reach maximum cell number.

When Luria and Delbrück assessed the variance (corrected for sampling) in the number of resistant bacteria in 8 small parallel cultures, this value ranged from a low of 40.8 to a high of 3,498 (their Table 2). In their Table 3 summarizing the results of two experiments, they showed that the plurality of cultures had zero resistant bacteria. In striking contrast, only one or a few cultures had hundreds of cells able to resist infection. The authors performed three experiments in each of which they took 10 samples from one large culture (their Table 1). They found the variances in the number of resistant bacteria were much lower for each of the three experiments: 15, 27, and 3.8.

**Figure 1.**
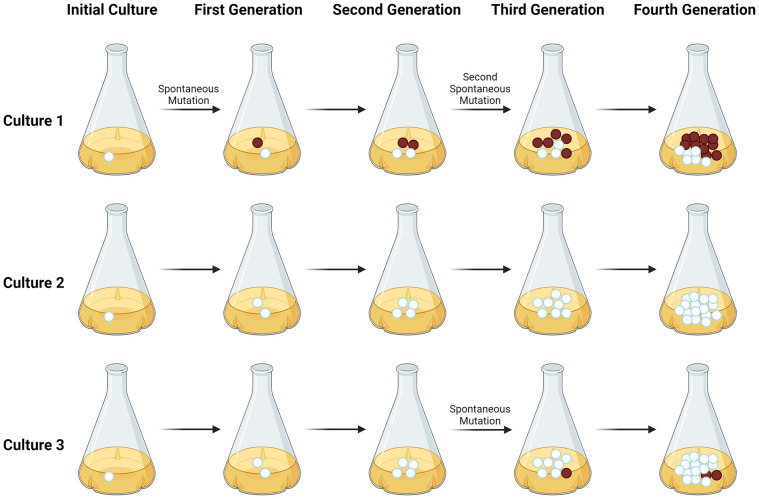
**The key principle behind the Luria-Delbrück assay is depicted above.** In short, if resistance is based on spontaneous and heritable mutations that occur independent of exposure to bacteriophage, as opposed to being induced by such exposure, parallel cultures should show great variation in the number of resistant cells present when the cultures reach maximum cell number. In each culture above, cells with resistance-conferring mutations are indicated by the color red. In Culture 1, where a mutation occurs in an early generation, the end culture will possess many cells carrying this mutation, resulting in a so-called “jackpot” of mutant cells. If, however, mutations occur in later generations of the culture (or not at all), as depicted in Cultures 2 and 3, the resultant culture will carry far fewer cells with resistance mutations. This diagram was created using BioRender and is based in part on Figure 1 in: Lang, G.I. Measuring mutation rates using the Luria-Delbrück fluctuation assay. *Methods Mol Biol*. 2018:1672:21-31.

This high magnitude of fluctuation in the number of resistant colonies among small separate cultures is logically entailed by the fact that mutations to resistance in early generations after culture initiation will lead to large numbers of resistant cells due to the exponential nature of cell growth. For example, if the culture were to involve 10 cell doublings, a mutation in the first such doubling would yield up to 1,024 resistant cells at the end of the culture, a so-called “jackpot” effect due to mutation in an early generation. If the first mutation to resistance occurred after 8 doublings, at the end of the culture there would only be 4 resistant cells. Figure 1 schematically illustrates this sort of variation in numbers of phage-resistant cells among parallel cultures based on where in the growth curve the mutations occur.

If, however, resistance arises, with some average probability per cell, through a mechanism dependent on and elicited by exposure to the virus, then the fluctuation in the numbers of resistant cells between cultures should be modest. This result is expected for this mechanism of resistance because the chances per cell for the phenotypic change to resistance should be constant across cultures. There is no “jackpot” effect as the virus is introduced only at the end of the cultures.

The results obtained by Luria and Delbrück were interpreted by them to represent the greater variation in numbers of resistant cells per culture predicted by the pre-exposure mutation hypothesis. As a result of that finding, Luria and Delbrück concluded that resistance to the bacteriophage used in these experiments (T1 phage), for this particular bacterial strain (*E. coli* B), resulted from spontaneous mutations independent of exposure to the virus. This study thus provided an early and dramatic illustration of the potential value of applying formal mathematical analysis to an experimental question in biology.

Luria and Delbrück's experimental approach was also an impressive example of what has been described as the hypothetico-deductive method that is elegantly described in the 1968 Jayne Lectures by Nobel Laureate Peter Medawar [4]. In this approach, the logical consequences of different hypotheses are worked out. If one can identify an experimental scenario in which the deductive consequences of different hypotheses differ significantly, then you have possibly identified an opportunity for a decisive experimental test.

It is worth noting that Luria and Delbrück stated that the resistant cells in this experimental system could no longer adsorb virus. This reproducible finding suggests, in light of what we now understand, that the predominant mutations they observed in their bacterial hosts were eliminating the biosynthesis of the receptor or altering its half-life or structure sufficiently to abrogate binding by the virus. In the context of current knowledge of the types of genetic or epigenetic mechanisms that can influence interactions between bacterial cells and the viruses that infect them, the loss of receptor binding is consistent with only a subset of such mechanisms, including mutations of the sort cited above.

Luria and Delbrück's conclusions were of fundamental importance not just for understanding the interactions between bacteria and their viruses but for biology and biomedicine more broadly. This study facilitated the development of the concept of random mutation, meaning that the mutation occurs through chemical mechanisms not directly influenced by the functional consequences of the mutation.

Furthermore, Luria and Delbrück were cautious in drawing inferences about the generality of their findings in that they explicitly acknowledge that their experiments characterized the mechanism of bacterial resistance to infection by a bacteriophage for one pairing of host cell and virus. They were also prescient in acknowledging (see quote directly below) that there might be other mechanisms operating in other bacteria infected by other types of phage even though the details of these pathways could not easily have been anticipated given the state of knowledge in 1943.

We consider the above results as proof that in our case the resistance to virus is due to a heritable change of the bacterial cell which occurs independently of the action of the virus. It remains to be seen whether or not this is the general rule. There is reason to suspect that the mechanism is more complex in cases where the resistant culture develops only several days after lysis of the sensitive bacteria. [1]

Of particular interest is the question of why the findings of Luria and Delbrück were so seemingly straightforward. In recent years, there has been intense interest in bacterial mechanisms that mediate immunity to bacteriophage, especially CRISPR-Cas systems, if only because they can be adapted for gene editing in humans and other species. If the bacteria used in the experiments of Luria and Delbrück had possessed a CRISPR-Cas resistance pathway for the phage (which they referred to as alpha phage but was later called T1 phage), the results should have been less clear-cut.

In that case, the observation correlating resistance with the absence of phage being able to bind to the bacteria should have been highly variable. Based on the preceding, it is of interest to note that, presumably by chance, the *E. coli* strain selected by Luria and Delbrück for their experiments lacked an operative CRISPR-Cas mechanism [5]. It has been estimated that only about half of bacteria possess CRISPR-Cas systems [6, 7].

Andrew Murray, an eminent cell and developmental biologist at Harvard, offered this assessment of the importance of the 1943 study by Luria and Delbrück:

The Luria–Delbrück article had three important impacts beyond its direct conclusion: it showed that elegant statistical analysis could illuminate biological processes that could not be directly observed, it contributed to Luria and Delbrück winning the 1969 Nobel Prize in Medicine or Physiology (shared with Alfred Hershey), and it led, indirectly, to a continuing debate about whether organisms exert physiological control over their mutation rates. [8]

Arguably, a fourth significant consequence of the Luria and Delbrück study is a methodology for determining microbial mutation rates. This methodology, revised in light of more recent insights, is still of value [9, 10].

While the conclusions of the Luria and Delbrück study of 1943 continue to be regarded as seminal contributions to biology and biomedicine, the finite nature of any inquiry cannot absolutely forestall the possibility that different experimental components, circumstances, or tools of analysis could yield insights not captured in any particular investigation. Just in terms of the mathematical analysis, in the intervening years, biomathematicians have refined understanding of mathematical distributions that better describe the mutational process than the one adopted by Luria and Delbrück [11].

In addition, since 1943, researchers, empowered by ever more sophisticated genetic and biochemical technologies, have discovered a number of parallel mechanisms for generating bacterial phenotypic variation that fosters survival in the context of bacteriophage infection or other threats, such as antibiotics. These evolutionary mechanisms of phenotypic change can be genetic (changes in nucleotide sequence), epigenetic (changes that do not involve alterations of nucleotide sequence but do influence phenotype by, for example, affecting the extent of gene transcription, messenger RNA translation, or protein stability), or involve elements that are difficult to classify as purely one or the other (mixtures of genetic and epigenetic changes) [12].

The new insights into the array of mechanisms that bacteria can use to fend off biological or chemical threats have sparked debate over the extent to which evolution is Darwinian or Lamarckian [12–14]. In this context, Darwinian evolution depends on phenotypic modification resulting from spontaneous changes in nucleotide sequence of one or more cellular genes that occur without being directly influenced by the nature of the change in phenotype. Lamarckian evolution or, more accurately, quasi-Lamarckian evolution is the result of heritable phenotypic change somehow caused by environmental influences without alteration of nucleotide sequences in the genes present prior to exposure to the environmental input. Mechanisms of resistance that depend on relatively stable changes in transcription, metabolism, or acquisition of new genetic material from sources outside the cell all represent pathways not addressed in the study from 1943.

While one can imagine different but reasonable characterizations of what Darwinian or Lamarckian evolutionary mechanisms are, further discussion and exploration of these complex concepts has the potential to enrich our understanding of the subtle ways evolutionary opportunism generates solutions to the challenge of survival and procreation. A fuller exploration of this topic is beyond the scope of our present focus. The reader is referred to references 12-14 as an introduction to this debate.
